# Machine Learning Approaches for Compound–Target Interaction Prediction: A Review

**DOI:** 10.3390/foods15091582

**Published:** 2026-05-04

**Authors:** Jingjie Zhang, Tengyu Li, Chi Yan, Yujue Li, Yonghui Yu, Jing Wang, Baoguo Sun

**Affiliations:** 1 Key Laboratory of Geriatric Nutrition and Health, Beijing Technology and Business University, Ministry of Education, Beijing 100048, China; 2 China-Canada Joint Lab of Food Nutrition and Health, Beijing Technology and Business University, Beijing 100048, China; 3 Key Laboratory of Special Food Supervision Technology for State Market Regulation, Beijing Technology and Business University, Beijing 100048, China

**Keywords:** machine learning, compound–target interactions, feature extraction, deep learning, neural networks, computational compound discovery

## Abstract

Compound–target interaction (CTI) prediction plays a critical role in drug discovery and the functional study of food-derived bioactive compounds. However, traditional experimental methods for CTI identification are limited by high costs, long cycle times, and high false-positive rates, highlighting an urgent need for more efficient approaches. Machine learning (ML) has become a revolutionary tool to address these challenges. In this review, we focus on recent developments in ML-based CTI prediction. We first systematically outline the commonly used public databases and feature extraction methods for both compounds (molecular fingerprints) and proteins (sequence-derived features), followed by elaborating on four types of ML approaches, including classical supervised learning, matrix factorization, graph topology-based inference, and deep neural network frameworks. In particular, this review explores the emerging application of these computational approaches in identifying targets of food-derived bioactive compounds, underscoring its significant potential to advance functional food research. Moreover, we analyze key challenges, such as limited model interpretability, high data dependency, and insufficient multi-source information integration, and put forth future prospects to improve the prediction of food-derived CTIs, thereby facilitating their application in functional food research.

## 1. Introduction

Compound–target interactions (CTIs) refer to the specific binding between bioactive molecules and their particular biological targets (especially biological macromolecules such as proteins). These molecules exert their pharmacological or health-promoting effects through this interaction [1,2]. Therefore, the prediction of CTIs is crucial not just in pharmaceutical drug discovery, allowing lead compound optimization, mechanism investigation, and drug repurposing, but also in the growing field of functional food research, which helps to understand the molecular targets of dietary bioactive components [3]. Traditional target screening methods mainly consist of high-throughput screening (HTS) and structure-based molecular docking techniques [4]. Over the past decades, despite making substantial contributions to pharmaceutical research, these methods have exhibited notable drawbacks, including high costs, lengthy development cycles, and a susceptibility to yielding false positives [5,6]. These limitations are evident in the economic statistics of modern drug development. The capitalized cost for each new drug averaged approximately $2.69 billion [7]. Moreover, the timeline from discovery to approval spanned 10~15 years [8], and the overall success rate from Phase I clinical trials to approval remained as low as 9.6% [9]. Such statistics showed that it is critical for more efficient strategies for CTI prediction.

**Figure 1:**
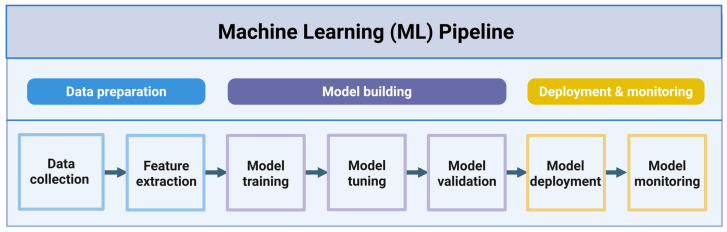
The comprehensive ML pipeline for CTI prediction. The workflow is organized into three primary phases: (1) data preparation, involving the collection of raw compound and target data from public databases followed by preprocessing and feature extraction; (2) model building, which encompasses model training, hyperparameter tuning, and performance validation using benchmark datasets; (3) deployment and monitoring, where the validated model is integrated into drug discovery workflows to predict novel interactions and continuously updated based on new experimental feedback.

Machine learning (ML) approaches have been widely employed with the rapid development of artificial intelligence technology. ML, as a core branch of artificial intelligence, enables computer systems to learn from data automatically and make predictions or decisions through algorithmic models [10]. In practice, a complete ML workflow typically involves several key stages, including data collection and preprocessing, feature extraction, model training and optimization, performance validation, prediction, and applications [11] (Figure 1). In contrast to conventional programming, ML mainly advances in its adaptive learning ability, robust generalization performance, and modeling complex nonlinear relationships with high efficacy [12] and has been gradually applied in diverse fields such as computer vision, natural language processing, and bioinformatics [13,14]. In the field of computer vision, a method aimed at addressing the limitations of existing anatomical structure detection methods was proposed. This approach used deep reinforcement learning and multi-scale image analysis techniques to integrate the appearance modeling of anatomical structures with the target search process into a unified behavioral framework [15]. In addition, Wang et al. [16] provided a CNN-based approach for the process of identifying potential target genes, predicting miRNAs, visualizing the unique miRNA patterns, and validating genomes. This method enhanced the efficiency of gene data processing approaches based on bioinformatics.

ML methods have also been gradually adopted in drug discovery, especially in the prediction of CTIs, which have revolutionized the process of target identification and validation. In early work in this field, binary classification methods were primarily employed. Basically, this approach provided a clear “yes/no” prediction framework by using both positive and negative interaction pairs [17]. The network inference and matrix factorization techniques were subsequently proposed to better analyze the network relationships and latent patterns [18]. The continuous development of ML, coupled with the explosive growth of biomedical data, has been driving the deep learning revolution. Models such as CNNs and graph neural networks (GNNs) have been proven highly effective at automatically learning layered features from raw molecular structures and protein sequences [19]. Recently, Transformer-based models were introduced into the prediction of CTIs. This approach used self-attention mechanisms to pick up on long-range dependencies effectively in both sequential and graph-structured data [20]. At the same time, the development of public databases like DrugBank [21], ChEMBL [22], and Universal Protein Resource (UniProt) [23] have provided rich, high-quality datasets, which are essential for building these reliable computational models. Furthermore, we have seen the great progression in feature extraction, which has moved from basic substructure fingerprints to more advanced circular and pharmacophore-based ones, as well as advanced sequence and structure-based protein descriptors. These methods have progressed collaboratively with methodological developments promoting the enhancement of prediction accuracy and generalizability [24].

ML-based prediction of CTIs has been widely applied in areas such as drug and target discovery, lead compound identification, and drug repurposing. This has significantly increased the efficiency of drug development. For instance, a study presented DrugCLIP, a ML framework for predicting CTI interactions. The tool, through screening against the 5-HT2A receptor, successfully pinpointed eight active agonists from a group of 78 candidate molecules, with the most effective one demonstrating activity in the nanomolar range [25]. In another study, an ML-based tool called DTIP was developed to predict drug–target interactions, specifically for Alzheimer’s disease (AD). A lot of potential drugs for AD treatment were found via DTIP-based screening and validation analysis, like masitinib, quetiapine, and miconazole [26]. Despite these successful applications, existing reviews have primarily focused on pharmaceutical drug discovery and organized the literature by chronological order of algorithms [27,28,29]. The methodological development in this field, especially from a feature–model co-evolution perspective, remains largely unexplored.

Food-derived bioactive compounds, including polyphenols, flavonoids, saponins, and terpenoids, have gained increasing attention in functional food research for their potential to modulate physiological functions and promote health [30,31]. Unlike conventional drugs that typically target a single protein with high affinity, these compounds often exert their effects through multi-target, low-affinity interactions, contributing to complex biological outcomes like antioxidant, anti-inflammatory, and gut microbiota-modulating activities [32]. However, the molecular targets of most food-derived bioactive compounds remain largely unknown, which limits their further development and application in functional food research [33]. Therefore, efficient strategies are needed to systematically identify compound–target associations. Computational approaches, especially ML-based CTI prediction, offer a way to accelerate target identification and large-scale interaction mapping [34]. But its application to food-derived bioactive compounds still remains in the early stages, and few studies have systematically examined how the distinctive features of these compounds, such as high structural diversity, limited interaction data, and multi-target mechanisms, affect model performance or require methodological adjustments.

In this review, we discuss recent advances in ML-based CTI prediction, with a focus on key databases, feature extraction methods, and machine learning approaches. We then examine how these computational methods have been applied, or adapted, to food-derived bioactive compounds and highlight opportunities and challenges in using ML-based prediction to support functional food research.

## 2. Review Methodology

This review follows a narrative review approach, aiming to provide a structured and critical overview of machine learning methods for compound–target interaction prediction. To ensure a comprehensive and representative coverage of the literature, a systematic literature search was conducted in the Web of Science, PubMed, and Google Scholar databases. The search covered the period from January 2005 to December 2025, capturing the development of machine learning applications in this field over the past two decades.

The search strategy combined terms related to compound–target interactions, machine learning methods, feature extraction, and applications in functional food research. The following keyword combinations were used: “compound–target interaction” OR “drug–target interaction” OR “CTI” AND “machine learning” OR “deep learning” OR “neural network” OR “matrix factorization” OR “graph” OR “supervised learning” OR “functional food factors” OR “feature extraction”. Only articles published in English were considered.

Studies were included if they presented original research, comprehensive reviews, or significant conference proceedings on machine learning-based prediction of compound–target or drug–target interactions, with a focus on methodological development, feature extraction strategies, or model architectures. Studies that solely applied existing methods without methodological innovation, or that focused exclusively on molecular docking or molecular dynamics simulations without machine learning components, were excluded.

**Figure 2:**
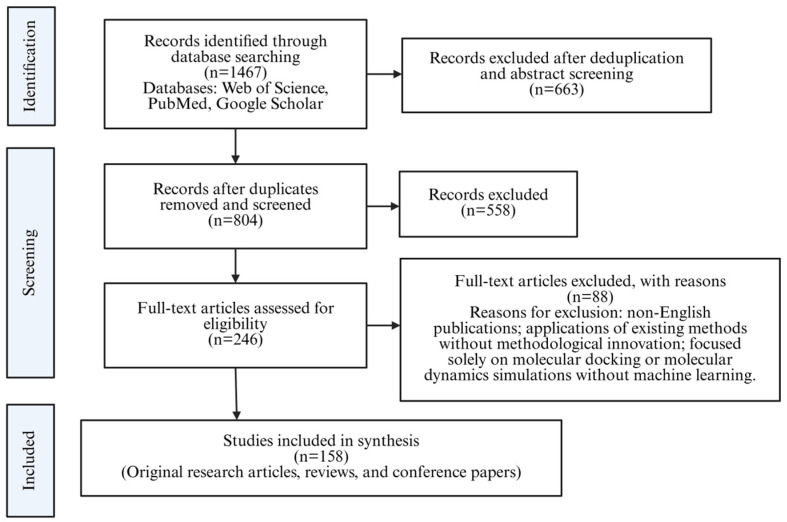
PRISMA-style flow diagram of the literature screening process. Records were identified from Web of Science, PubMed, and Google Scholar (*n* = 1467). After duplicate removal (*n* = 804), records were screened by titles and abstracts, with 558 records excluded. The remaining 246 full-text articles were assessed for eligibility; 88 articles were excluded (e.g., no methodological innovation, no ML component). Finally, 158 articles were included in the review.

The literature screening was conducted manually by two reviewers, with disagreements resolved through discussion. The initial search yielded 1467 articles. After removing duplicates, 804 articles were screened based on titles and abstracts. Of these, 246 articles were selected for full-text assessment, and ultimately 158 articles were included as the primary basis of this review, covering original research articles, reviews, and conference papers. The literature screening process is illustrated in Figure 2. The included literature spanned from 2005 to 2025, with most articles published in leading bioinformatics, cheminformatics, and computational biology journals. We showed preference to studies that introduced novel methodologies, provided benchmark comparisons, or addressed key challenges in the field, with special attention to those exploring applications to food-derived bioactive compounds.

## 3. Data Sources and Feature Representation

Accurate prediction of CTIs relies critically on the obtaining of high-quality data and the development of effective feature representation methods. In this section, we systematically introduce the data resources and feature extraction strategies that are commonly employed in CTI prediction research.

### 3.1. Data Resources

The quality and scope of data resources directly shape model performance and reliability, making data the bedrock of ML-based CTI prediction. Over the years, researchers have developed a range of databases to support this task. These can be broadly grouped into six categories based on their primary focus: (1) comprehensive pharmaceutical databases, (2) CTI databases, (3) protein and biomolecular databases, (4) chemical compound information databases, (5) food ingredients related databases, and (6) other databases. Together, they provide not only the positive and negative sample pairs needed for model training but also the structural information of compounds and the biological characteristics of targets, essentially laying the groundwork for CTI prediction (Table 1).

#### 3.1.1. Comprehensive Pharmaceutical Databases

Comprehensive pharmaceutical databases such as DrugBank, Kyoto Encyclopedia of Genes and Genomes (KEGG) DRUG, and ChEMBL integrate multidimensional information, including drug chemical structures, pharmacological parameters, target biological data, and clinical applications, but each has its own emphasis. DrugBank [21] stands out for its detailed interaction mechanisms and structured data fields, making it well suited for mechanism-driven CTI studies. KEGG DRUG [35] organizes drugs by therapeutic class and Anatomical Therapeutic Chemical (ATC) codes within the KEGG pathway framework, which is particularly useful for systems pharmacology approaches that consider interactions in a broader biological context. ChEMBL [22] contains over 5.4 million experimentally measured bioactivity data points, covering binding affinities, functional activities, and ADMET properties, offering the scale needed for data-intensive machine learning applications. Together, these databases provide complementary resources for different modeling strategies.

#### 3.1.2. CTI Databases

CTI databases focus specifically on validated interactions and binding data mainly including Search Tool for Interacting Chemicals (STITCH), BindingDB, SuperTarget and Manual Annotated Targets and Drugs Online Resource (MATADOR). STITCH [36] integrates experimentally validated and text-mined chemical–protein interactions with confidence scores. By incorporating text-mined data from scientific literature, it includes interactions that may not yet have been experimentally validated, which makes it particularly valuable for studying less-characterized compounds such as dietary bioactive substances, where experimental interaction data are often sparse. For instance, STITCH has been used to explore interactions between natural compounds from plants like *Satureja nepeta* and inflammatory targets such as IL-6 [37]. BindingDB [38], in contrast, focuses on quantitative precision. It provides over 1.1 million experimentally validated binding constants (Kd, Ki) across more than 7000 target proteins, making it a primary resource for machine learning models that rely on binding affinity as the prediction target. SuperTarget [39] and MATADOR [40] take a different direction by adding contextual layers. SuperTarget links interactions to adverse effects and drug–target–disease relationships, while MATADOR provides manually curated associations with regulatory information. These features make them suitable for network-based studies, such as drug repurposing or exploring polypharmacology, an area relevant to food-derived compounds that often act through multiple targets. Taken together, these databases offer complementary resources, including coverage breadth and text mining capacity (STITCH), quantitative accuracy (BindingDB), and contextual annotations (SuperTarget and MATADOR), thus supporting the development and evaluation of ML models for CTI prediction.

#### 3.1.3. Protein and Biomolecular Databases

Protein and biomolecular databases, such as Universal Protein Resource (UniProt), Braunschweig Enzyme Database (BRENDA), and Biological General Repository for Interaction Datasets (BioGRID), serve a different role in CTI prediction. They provide essential information for target characterization. UniProt [23] offers comprehensive protein sequence and functional annotation, including subcellular localization and post-translational modifications. BRENDA [41] complements this by compiling enzyme kinetic parameters from over 140,000 scientific papers, which is particularly relevant when predicting interactions involving metabolic enzymes. Pfam [42] uses hidden Markov models to classify protein families and domains, helping to generalize predictions across related proteins, especially when dealing with understudied targets. BioGRID [43], with its curated collection of protein–protein interactions (PPIs), genetic interactions, and post-translational modification data, supports network-based analyses that can place predicted CTIs within broader cellular pathways.

#### 3.1.4. Chemical Compound Information Databases

Chemical compound information databases focus on small molecules. PubChem [44] is one of the largest public repositories, containing structural, physicochemical, and bioactivity data for over 119 million compounds. Its sub-database, BioAssay, offers standardized high-throughput screening data that can be used as positive or negative interaction evidence in CTI model training. ZINC-22 [45] complements PubChem by focusing on commercially available molecules, providing three-dimensional structures for over 200 million compounds and supporting virtual screening and substructure searches. While these databases are not CTI-specific, they supply the structural data needed for compound feature extraction and for retrieving candidate molecules in prediction tasks.

#### 3.1.5. Food Ingredients-Related Databases

The Food Database (FooDB) and the Traditional Chinese Medicine Systems Pharmacology Database and Analysis Platform (TCMSP) also play important roles in CTI prediction, with special value in the prediction of targets for food-derived bioactive compounds. FooDB serves as the most comprehensive online food ingredient database, containing chemical and biological data for over 70,000 food components. It has been increasingly used in computational target prediction. For instance, PhyteByte, a machine learning-based tool, used FooDB compounds and ChEMBL activity data to predict food-derived compounds that interact with specific protein targets [46]. Similarly, the PhyteByte tool leveraged FooDB as the compound source and employed a random forest classifier with molecular fingerprints to predict interactions between food-derived compounds and specific protein targets [47]. TCMSP [48] integrates herbal ingredients, targets, and pharmacokinetic parameters for traditional Chinese medicine research. These databases provide resources for studies involving natural products, combination therapies, and safety assessment. It has been widely employed in network pharmacology and CTI prediction. For example, a deep learning framework (MMFi-DPBML) integrating multi-molecular fingerprint features was developed to predict ingredient–target interactions in TCM using TCMSP data, demonstrating that structural and spatial features could be effectively combined for accurate prediction [49]. Another study used TCMSP to predict anti-SARS-CoV-2 compounds via ML methods, with six predicted compounds subsequently validated in experimental assays [50].

However, both databases have less comprehensive interaction annotations compared to pharmaceutical databases like DrugBank or ChEMBL, which limits their direct use for training large-scale ML models. Therefore, food-focused CTI prediction often requires combining these specialized resources with more general databases or employing transfer learning from pharmaceutical data.

#### 3.1.6. Other Databases

Beyond the categories above, several other databases address specialized areas such as clinical pharmacology, drug safety, and combination therapy. Side Effects Database (SIDER) and FDA Adverse Event Reporting System (FAERS) focus on drug safety. SIDER [51] provides data on adverse drug reactions for approximately 6000 drugs, while FAERS collects real-world adverse event reports from the FDA. Drug Combination Database (DCDB) [52] compiles drug combinations and their mechanisms, supporting research on polypharmacy and synergistic effects. These databases provide resources for studies involving drug safety assessment and combination therapy.

Together, these databases extend the scope of CTI prediction beyond single drugs and well-studied compounds, supporting studies that span from molecular mechanisms to system-level analysis—covering synthetic drugs, natural products, and food-derived bioactive compounds.

### 3.2. Feature Extraction

As a key preprocessing step in ML-based CTI prediction, feature extraction converts the original biomedical data into a format suitable for machine learning models. Compounds and targets require different types of representations: molecular fingerprints for small molecules and sequence-based features for proteins, each with its own way of capturing relevant information. The choice of representation directly affects model performance, and a variety of methods have been developed over the years to handle different aspects of this task.

#### 3.2.1. Compound Molecular Fingerprints

Molecular fingerprints convert molecular structures into fixed-length bit strings or vectors. The underlying principle is to encode the presence, absence, or frequency of specific substructures within a molecule. Several fingerprint types have become standard in the field, including substructure-based, circular, path-based, and pharmacophore-based fingerprints. Each was developed with different priorities in mind, such as interpretability, computational efficiency, or sensitivity to structural variation. As the focus of computational chemistry has shifted toward tasks such as CTI prediction, these fingerprints have increasingly moved beyond capturing generic structural features toward encoding properties more directly relevant to biological activity (Table 2).

1. Substructure-based Fingerprints

Substructure-based fingerprints encode molecular structures as a binary string, where each bit indicates the presence or absence of a predefined fragment. In cheminformatics, common substructure-based fingerprints, such as molecular access system (MACCS) bonds, PubChem fingerprints and Klekota-Roth fingerprints, have been commonly employed for tasks including CTI prediction. In practice, each fragment in a predefined dictionary corresponds to a specific bit: set to 1 if the fragment occurs in the molecule and 0 otherwise. A notable strength of this approach is its interpretability. Because each bit maps directly to a known structural fragment, it is possible to trace a prediction back to specific chemical features [62]. This property makes substructure-based fingerprints particularly suitable for rule-based screening and for applications where understanding the basis of a prediction is essential.

However, the fixed dictionary that enables interpretability also imposes a fundamental limitation. Any structural features that are not included in the dictionary, such as novel, complex or simply unexpected structures, are effectively invisible to the representation [63]. As a result, these fingerprints lose fidelity when applied to compounds outside the predefined chemical space. Thus, substructure-based fingerprints are most appropriate when interpretability takes precedence and the compounds under investigation fall within well-characterized chemical territory. For tasks involving novel scaffolds or structurally diverse natural products, other fingerprint types tend to perform better.

2. Circular Fingerprints

Unlike substructure-based fingerprints that rely on predefined dictionaries, circular fingerprints, also known as topological fingerprints, are designed to capture local chemical environments around each atom without using a predefined fragment dictionary. Common algorithms include Extended Connectivity Fingerprints (ECFP) and the Morgan fingerprint, as implemented in RDKit. For each non-hydrogen atom, these methods expand outward to include neighboring atoms within a specified radius [64]. The resulting circular substructures are then hashed into a fixed-length fingerprint. This approach automatically captures structural features relevant to biological activity, many of which would not be pre-encoded in a dictionary. Consequently, circular fingerprints often achieve strong predictive performance in bioactivity prediction and virtual screening. They are also robust for minor structural variations, such as bond order changes or small substituent shifts.

The trade-off, however, is reduced interpretability. Unlike substructure-based fingerprints, it is difficult to trace which specific atomic environment contributed to a given prediction, making the model more of a “black box”. Nevertheless, because of their consistently high predictive power, circular fingerprints have become the de facto standard in quantitative structure–activity relationship (QSAR) modeling and are particularly well suited for large-scale screening tasks where predictive accuracy is prioritized over direct mechanistic explanation. Circular fingerprints, especially ECFP and its variants, are widely used in compound–target prediction. They have been applied to a range of tasks, such as drug design [65], target prediction [66], and binding affinity prediction [67].

3. Path-based Fingerprints

Path-based fingerprints were developed to capture molecular backbone structures and connectivity patterns by enumerating all linear paths of a defined length within a molecule. Each unique path is hashed and mapped to a fixed position in the fingerprint, with the Daylight fingerprint being the classic implementation of this approach [2]. This representation excels at encoding molecular skeletons and overall topology, making it particularly effective for molecular similarity evaluation and scaffold analysis. In drug discovery, these properties are especially valuable during lead optimization, where the goal is to modify peripheral substituents while preserving the core scaffold. For instance, the ErG (Extended Reduced Graph) fingerprint was specifically developed to outperform Daylight fingerprints in scaffold hopping tasks; in a benchmark across 11 activity classes from the MDL Drug Data Report database, ErG performed as well as, or better than, Daylight fingerprints in 10 cases, demonstrating that specialized representations can offer advantages over general purpose path-based fingerprints for certain scaffold hopping applications [68].

The main limitation of path-based fingerprints is their reduced sensitivity to detailed atomic environments. They treat each path independently and may miss global structural context or subtle electronic effects that can be crucial for binding specificity [69]. As a result, they are less discriminative than circular fingerprints for distinguishing closely related compounds that differ primarily in local chemistry rather than overall connectivity.

Thus, path-based fingerprints are most suitable when the primary objective is to identify compounds with similar core structures, such as in scaffold hopping campaigns or when assessing molecular similarity across a series of analogs [70]. For tasks where local chemical environments or functional group details dominate the interaction, circular or pharmacophore-based fingerprints typically provide better predictive performance [71].

4. Pharmacophore-based Fingerprints

Pharmacophore-based fingerprints were developed to capture the functional features that directly mediate molecular recognition, rather than encoding atoms or bonds directly. This approach abstracts a molecule into a set of key interaction elements, including hydrogen bond donors and acceptors, hydrophobic centers, charged groups, and aromatic rings, along with their spatial relationships in two or three dimensions [72]. By focusing on the chemical features that drive binding, pharmacophore fingerprints can identify molecules with similar biological activity, even when their core skeletons are entirely different. This property makes them particularly valuable in virtual screening and target deconvolution, especially for natural products and food-derived compounds, where diverse scaffolds often converge on the same target [73].

The main limitation of pharmacophore-based fingerprints lies in their dependence on accurate feature assignment and, for three-dimensional pharmacophores, on conformation generation. Different conformations can yield different pharmacophore models, introducing variability and computational overhead. Additionally, they abstract away detailed structural information that may be relevant for binding specificity. Thus, pharmacophore-based fingerprints are most effective when the goal is to discover structurally novel compounds that share a common binding mode with known actives. They are particularly well suited for screening natural product libraries or food-derived compound collections, where scaffold diversity is high. For tasks requiring fine-grained structural discrimination among closely related analogs, topology-based fingerprints such as circular or path-based methods often perform better.

When dealing with food-derived compounds, which often exhibit high structural diversity and complex natural product scaffolds, the choice of fingerprint becomes critical. Substructure-based fingerprints are interpretable but may miss novel scaffolds not present in their predefined dictionaries. Circular fingerprints are robust to structural variations and have been successfully applied to predict targets of dietary polyphenols and flavonoids [66]. Pharmacophore-based fingerprints are particularly suitable for scaffold hopping, enabling the identification of structurally diverse food compounds that share the same biological target [73]. Path-based fingerprints excel at molecular skeleton comparison, which is useful for assessing similarity among food-derived analogs. For instance, a benchmarking study comparing multiple fingerprint methods for natural product screening found that ECFP consistently outperformed substructure-based fingerprints and that combining different fingerprint types further improved the screening performance [74].

#### 3.2.2. Target Protein Feature Extraction

Protein feature extraction converts amino acid sequences into numerical descriptors suitable for ML models. The underlying principle is to transform sequence information into a fixed-dimensional representation while preserving biologically relevant properties, such as composition, physicochemical characteristics, or evolutionary signals. Several protein representation types have become standard in the field, including sequence-based, structure-based, evolution-based, and deep learning-based features. Each was developed with different priorities in mind, such as computational efficiency, structural detail, evolutionary conservation, or representation learning capacity. As the focus of CTI prediction has shifted toward tasks such as binding affinity estimation and cold-start target discovery, these representations have increasingly moved beyond capturing generic sequence statistics toward encoding features more directly relevant to CTIs (Table 3).

1. Sequence-based Features

Sequence-based features are derived directly from the linear amino acid sequence of a protein. The underlying principle is to compute statistical or physicochemical properties from the sequence without requiring structural or evolutionary information. Common descriptors in this category include amino acid composition, dipeptide and tripeptide composition, pseudo amino acid composition (PseAAC), and various physicochemical property profiles [81]. A notable strength of sequence-based features is their computational efficiency. Because they only need the primary sequence as input, they can be generated quickly for large numbers of proteins, making them suitable for high-throughput screening scenarios or when only sequence information is available.

However, these features ignore higher-order structural and evolutionary information. Binding often depends on three-dimensional conformation or conserved functional residues, which sequence-based descriptors do not capture. Consequently, their predictive accuracy can be limited for targets where binding is conformation-dependent or for proteins with no close homologs [82].

Thus, sequence-based features are most appropriate when computational resources are limited, when only sequence data are available, or as a baseline for more sophisticated representations. For tasks requiring structural or evolutionary insight, structure-based or evolution-based features typically offer better predictive performance.

2. Structure-based Features

Structure-based features are derived from the three-dimensional conformation of a protein, capturing spatial and geometric information that is not available from the linear sequence alone [83]. The underlying principle is to encode structural properties such as secondary structure elements, solvent-accessible surface area, surface charge potential, and binding pocket geometry into numerical descriptors [84]. A key advantage of structure-based features is their direct relevance to molecular recognition. Binding interactions depend on the precise arrangement of atoms in three-dimensional space, and these features explicitly represent the physical and chemical properties of the binding site. This makes them particularly valuable for structure-based drug design, for understanding detailed binding mechanisms, and for tasks such as docking or binding affinity prediction.

The main limitation is that high-quality structural information is not available for all proteins. Experimental structures determined by X-ray crystallography or NMR exist for only a fraction of known targets. Computational prediction methods, such as AlphaFold, have expanded coverage, but they introduce additional complexity and potential inaccuracies, especially for proteins with no close structural templates [85].

Thus, structure-based features are most suitable when reliable three-dimensional structures are available or can be confidently predicted. They are particularly valuable for targets with well-characterized binding pockets or for mechanistic studies where spatial information is essential. When structural data are lacking or of low confidence, sequence-based or evolution-based features may be more practical alternatives.

3. Evolution-based Features

Evolutionary conservation often signals functional importance. Residues critical for ligand binding, catalytic activity, or protein stability tend to be preserved across homologous sequences, while variable regions may tolerate change. Evolution-based features exploit this principle by capturing conservation patterns derived from multiple sequence alignments. Position-Specific Scoring Matrices (PSSMs) [86] and Hidden Markov Model (HMM) profiles [87] are two widely used methods that quantify which positions in a protein family are highly conserved and which are divergent. These features are particularly valuable when three-dimensional structures are unavailable. Unlike structure-based representations that require experimental or predicted coordinates, evolution-based features can be generated from sequence data alone while still providing functional insight. In CTI prediction, they have been shown to improve binding site detection and interaction modeling, especially for targets with many known homologs [88]. They also support transfer learning, where a model trained on a well-characterized family can be applied to related but less-studied proteins.

The main drawback is data dependency. Building reliable evolutionary profiles requires a sufficient number of diverse homologous sequences. For understudied proteins, such as many food-derived targets or proteins from non-model organisms, the available sequence data may be too sparse to construct informative alignments. Additionally, generating PSSM or HMM profiles involves searching large databases, which is computationally more intensive than simple sequence-based features.

In practice, evolution-based features work best when the target belongs to a well-represented family with many known homologs. They offer a middle ground between fast but shallow sequence-based features and data hungry deep learning representations. When homologs are scarce, sequence-based or deep learning-based features may be more practical choices. For example, evolutionary inference has been successfully applied to predict the functions of understudied protein kinases and pseudokinases, enabling functional annotation in the “dark” proteome where traditional methods often fail [89].

4. Deep Learning-based Features

Instead of relying on hand-crafted rules or statistical summaries of sequences, deep learning-based features learn representations directly from large-scale protein sequence data. Models such as ProtTrans [90] are pre-trained on millions of protein sequences using self-supervised objectives, producing dense vector embeddings that capture both local and global patterns. CNNs and RNNs, including LSTMs, have also been used to extract features from sequences, with CNNs identifying short motifs and RNNs modeling long-range dependencies [91,92]. What sets deep learning-based features apart is their capacity to learn hierarchical representations without manual feature engineering. They implicitly encode structural, evolutionary, and functional information, often achieving state-of-the-art performance on tasks such as binding affinity prediction and remote homology detection. Once pre-trained, these models can be fine-tuned for specific CTI prediction tasks with relatively limited labeled data.

Deep learning models are data hungry during pre-training, requiring large, diverse sequence corpora. They are also computationally expensive to train from scratch, though pre-trained embeddings can be used as off-the-shelf features [93]. Interpretability remains a challenge; it is not always straightforward to determine which parts of a sequence drove a given prediction, although attention mechanisms and saliency maps offer some insight [94].

These features are most advantageous when predictive performance is the top priority and sufficient unlabeled sequence data are available for pre-training or when a well-trained model can be fine-tuned. For smaller-scale studies or when computational resources are limited, simpler sequence-based or evolution-based features may be more practical. In recent years, hybrid approaches that combine deep learning embeddings with traditional features have gained traction, leveraging the strengths of both paradigms. For example, in a benchmarking study comparing 12 state-of-the-art deep learning architectures, DeepConv-DTI consistently outperformed other models across the majority of CTI prediction datasets. This method combines sequence-based protein features with convolutional neural networks and achieved an MCC of 0.6 or higher while remaining computationally efficient [95].

In CTI prediction, some target proteins, especially those that interact with food sources or those from non-model organisms, may be understudied. For such proteins, the lack of diverse homologous sequences limits the reliability of traditional evolution-based features (e.g., PSSM). Therefore, specialized informatics tools, including advanced evolutionary inference and protein language models, are increasingly used to annotate these targets and predict their functions [89]. Sequence-based features provide a fast baseline but fail to capture functional binding sites. Structure-based features are ideal when high-quality 3D structures are available, but many food-related targets lack experimental validation. Deep learning-based features, such as ProtTrans embeddings, offer a promising alternative: pre-trained on millions of sequences, they can generalize understudied proteins and have been used to improve binding affinity prediction for food-derived peptides [94]. In practice, a hybrid approach combining deep embeddings with traditional features often yields the best performance for food-related CTI prediction.

## 4. ML-Based Methods for CTI Prediction

**Figure 3:**
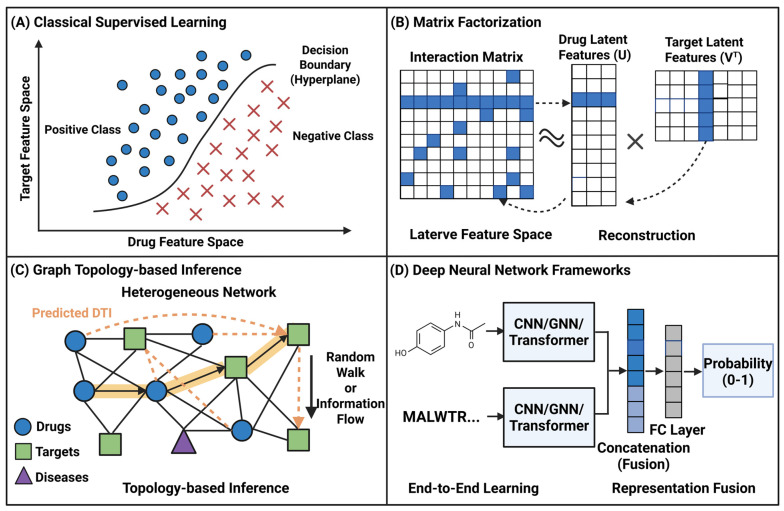
Comparison of four mainstream ML paradigms for CTl prediction. (**A**) Classical supervised learning: treats CTl prediction as a supervised classification task separating positive and negative pairs using a decision boundary. (**B**) Matrix factorization: maps compounds and targets into a shared low-dimensional latent space to reconstruct the interaction matrix and fill missing entries. (**C**) Graph topology-based inference: constructs heterogeneous networks integrating multi-source data and infers novel interactions via topology-based methods like random walk. (**D**) Deep learning: employs neural network architectures (e.g., CNN, GNN, and Transformer) to automatically learn high-level representations from raw molecular data (SMILES, sequences) for end-to-end prediction.

In recent years, a variety of ML-based calculation methods have been developed to predict CTIs. According to their algorithm paradigm and historical evolution, we divide these methods into four types: (1) classical supervised learning, which relies on hand-crafted features; (2) matrix factorization, based on potential factor analysis; (3) graph topology-based inference, focusing on network communication; and (4) deep neural network frameworks, end-to-end representing the modern paradigm of learning (Figure 3; Table 4).

### 4.1. Classical Supervised Learning Methods

In CTI prediction, classical supervised learning methods usually describe the problem as a binary classification task. Each data point, such as compound–target pairs, is assigned to one of two mutually exclusive categories, usually called positive or negative classes. As discrimination models, their performance largely depends on feature representations, including molecular fingerprints and protein descriptors. As discussed in Section 2, these features are used to learn decision-making boundaries in feature space [17]. Models such as Support Vector Machines (SVMs) and Nearest Neighbor algorithms have been widely used in this task [96,97]. These classifiers effectively simplify complex interaction patterns into binary “yes/no” decisions through constructed feature vectors, and their performance is usually evaluated using indicators such as the Area Under the Precision–Recall Curve (AUPR) [98]. This intuitive decision-making logic, combined with the transparent interpretation of the contribution of characteristics, makes the method highly interpretable. Therefore, the prediction is not only accurate but can also provide mechanistic insights that can guide the subsequent experimental design.

The early work of CTI prediction mainly focuses on the development of feature engineering and traditional classifiers. For instance, Yamanishi et al. [6] solved the problem by presenting it as a binary classification task in the supervised binary diagram learning framework. This framework incorporated heterogeneous data sources, including chemical structures and pharmacological information, as input features. The AUC of their model on enzyme, ion channel, GPCR and nuclear receptor reached 0.904, 0.851, 0.899 and 0.843, respectively. Bleakley et al. [99] proposed the Bipartite Local Model (BLM) by introducing the decoupling feature extraction strategy, which expanded this research direction. Unlike holistic approaches, BLM extracted features from compounds and proteins, respectively, then built a local prediction model for each compound and target node in the diagram, so that the method can capture the specific patterns of each node in the network. Therefore, BLM has achieved higher AUPR scores on four benchmark datasets: 0.841 (enzyme), 0.813 (ion channel), 0.667 (GPCR) and 0.612 (nuclear receptor). Despite its high effectiveness in CTI prediction, BLM’s pure local learning design confined it to the direct network neighborhood of each node. Consequently, it struggled with compounds or targets that lacked existing interaction data, and this made novel target discovery particularly challenging. In order to solve this limitation, the follow-up research focused on enhanced feature representation strategies. Mei et al. [100] improved the BLM by proposing a neighbor-based interaction profile inference method, and their model’s AUC on enzymes, ion channels, GPCR and nuclear receptors reached 0.988, 0.990, 0.984 and 0.981, respectively. To further enrich the feature space, Mousavian et al. [101] used PSSM to extract protein features. Their methods reached 0.948, 0.889, 0.872 and 0.869, respectively, for the subserve area (AUC) of enzymes, ion channels, GPCR and nuclear receptors in four benchmark databases. Ding et al. [96] constructed their model by integrating molecular fingerprints, multivariate mutual information and network topological features. The model performed better in most target categories, with an AUPR score of 0.899 for enzymes, 0.929 ion channels, and 0.821 for GPCR, but fell to a 0.655 increase and decrease in nuclear receptors.

In binary classification, balanced samples are essential for building a robust model. Models can often learn effectively under balanced data, and a number of problems will arise when the data are unbalanced, including prediction deviation, performance index distortion, and decline in the ability to handle unbalanced categories. As the verified interactive data become richer, the lack of reliable negative samples emerged as a critical bottleneck. This scarcity severely limited the performance of computational prediction methods, thus promoting the development of various techniques to solve the issue. Liu et al. [102] constructed a set of negative samples with high confidence based on a clear logical premise. They argued that, if the similarity of a protein to any known or predicted compound target was low, it was unlikely to interact with the compound. Ezzat et al. [103] adopted an integrated method to solve two types of category imbalances. They evenly sampled negative instances in the base learner to solve the imbalance between classes and oversampled positive instances after clustering to deal with intra-class imbalances. This dual strategy allowed the model to learn from a complete range of the majority of data without ignoring the rare few patterns, thus improving the performance of CTI prediction. Keum et al. [97] used clustering to discover potential interactions between unknown pairs. Then, they combined the BLM framework with the self-training SVM to iteratively train unlabeled samples and finally obtained an optimized local classifier.

Binary classification methods used for CTI prediction have developed significantly in recent years. The field has moved away from relying on single models to more diversified technologies and new frameworks. This transformation has particularly manifested in three key trends: the deep integration of feature engineering and advanced ML models, the expansion from simple binary judgment to multi-label classification, and the paradigm conversion integration of semi-supervised learning and knowledge atlas, all of which transcend the traditional classification framework. Meng et al. [104] proposed a new model called Predicting Drug Targets with Protein Sequence (PDTPS) that integrated binary probability, PSSM, principal component analysis, and correlation vector machine. They ran five-fold cross-validation on enzymes, ion channels, GPCRs, and nuclear receptors, and the results were impressive, with average accuracies of 97.73%, 93.12%, 86.78%, and 87.78%, respectively. Chu et al. [105] proposed a community detection method, DTI-MLCD, which enhanced performance through multi-label classification strategies and added new positive CTI sample data. Tanoori et al. [106] proposed a semi-supervised transfer learning approach that reframed CTI prediction as a continuous binding affinity estimation task, thereby bypassing the limitations of discrete binary classification.

Classical supervised learning methods have played a foundational role in CTI prediction, turning complex problems into well-defined learning tasks. However, despite considerable efforts in optimizing feature engineering, sample balancing, and model integration, these methods continue to face persistent challenges in areas such as the “cold-start” prediction for novel targets/compounds, effective utilization of high-dimensional sparse features, and the deep integration of domain knowledge into the models. Compared to later approaches, classical supervised learning remains valuable when interpretability is paramount and datasets are small. However, their reliance on hand-crafted features limits their ability to capture complex nonlinear interaction patterns, and they often struggle when faced with sparse interaction data. This limitation created an opportunity for matrix factorization methods, which subsequently demonstrated significant advantages in handling data sparsity and cold-start scenarios.

### 4.2. Matrix Factorization Methods

In the field of CTI prediction, matrix factorization methods break down a large, sparse interaction matrix into smaller matrices containing latent features, providing a data-driven way to automatically learn important features [107]. This approach maps compounds and targets into a shared latent semantic space, where molecules and proteins with similar interaction patterns obtain closely aligned vector representations through minimizing the reconstruction error of known interactions [108]. Instead of relying on manually designed descriptors, this approach captures the hidden topological structures within known interaction data, allowing it to infer unknown interactions more effectively [109]. Then, the trained model can predict novel interactions by comparing the similarities between these learned latent vectors. Therefore, this approach serves as a robust computational framework for large-scale compound–target screening, especially in the presence of data sparsity.

Early work in this area projected chemical and genomic data into a unified “pharmacological space”, establishing the foundation for applying matrix factorization approaches in CTI prediction [6]. Later research improved the stability of the model by combining the probability model with the principle of collaborative filtering so as to effectively integrate diversified data from multiple sources. For example, Gönen et al. [110] proposed the Kernelized Bayesian Matrix Factorization (KBMF) method for predicting CTIs. In this method, they combined a Bayesian probability framework with multi-core learning, which not only effectively reduces data noise and uncertainty but also systematically integrates a variety of similarity measures of compounds and targets, including chemical structure similarity and sequence similarity. In the evaluation on four benchmark datasets, KBMF achieved AUC scores of 0.832, 0.799, 0.857, and 0.824 for enzymes, ion channels, GPCRs, and nuclear receptors, respectively.

Subsequent research expanded on basic matrix factorization methods across multiple dimensions. Zheng et al. [109] developed the Multiple Similarities Collaborative Matrix Factorization (MSCMF) method, which aimed to solve the integration challenge brought about by the heterogeneity of compound and target data by fusing multiple compound and target similarity matrices. The key was that the weights of these matrices were learned directly from the data, and the similarity that can best improve the prediction performance was automatically selected. On four benchmark databases, MSCMF achieved AUPR scores of 0.894 (enzymes), 0.937 (ion channels), 0.773 (GPCRs), and 0.673 (nuclear receptors). Liu et al. [111] further proposed the Neighborhood Regularized Logistic Matrix Factorization (NRLMF) algorithm. This method focused on modeling the probability of CTI through logistic matrix factorization, using compounds and targets with compound-specific and target-specific latent vectors, respectively, and incorporated neighborhood information for regularization. The method not only captured the collection pattern between similar candidates but also preserved the correlation topology between the chemical spaces and the target spaces, which effectively alleviated the problem of data sparseness and enhanced the robustness of prediction. It attained AUC scores of 0.966, 0.964, 0.930, and 0.851 on the same four target classes across benchmark databases. In addition, Ezzat et al. [112] proposed two graph-regularized matrix factorization methods to solve the cold-start problem when predicting the interaction of new targets. Their method aimed to build similarity-based graphs and then constrain the potential representations learned to reflect the structure of these graphs. By enforcing this constraint, the model could effectively spread the interactive information of the known target to the new target. Therefore, the model could still generate reliable predictions even for targets without previous interaction records.

Data sparseness and optimization difficulties are two persistent challenges in matrix decomposition models. In order to solve these difficulties, Zhang et al. [113] developed iPALM-DLMF, which is a method to frame CTI prediction as a non-negative matrix factorization problem. By introducing graph double-regularization and L2,1 normal regularization, their methods effectively reduced the sparseness of features and improved the convergence of the model. In a complementary approach, Xu et al. [114] proposed ConvBLS-DTI, a framework that combined a convolutional broad learning system with neighborhood regularized logic matrix factorization for CTI prediction. The method first used the matrix factorization component to extract meaningful features from the data and then used the generalized learning system to deal with data sparseness and incompleteness more effectively. In summary, these studies not only provided new solutions to the sparseness and scalability challenges in CTI prediction but also broadened the methodological framework of matrix decomposition.

In traditional matrix factorization models, the relationship is captured by the linear combination of potential features, while the interaction in the real world is usually more complex. In order to overcome this limitation, recent methods have turned to neural networks, which are better at modeling complex nonlinear patterns hidden in data, which has brought significant improvements to CTI prediction. In the early stages of combining deep learning with matrix factorization, the research mainly focused on using deep learning techniques to enhance feature representation or optimize objective functions within the matrix factorization framework. Xia et al. [115] applied a method based on the chemical genome to efficiently identify potential CTIs and proposed a method called self-paced learning with collaborative matrix factorization (SPLCMF) based on weighted low-rank approximation. Ding et al. [116] introduced a triple collaborative matrix factorization model with multiple kernels, achieving AUPR scores of 0.912, 0.933, 0.752, and 0.552 for enzymes, ion channels, GPCRs, and nuclear receptors, respectively, on four benchmark databases. Sajadi et al. [117] successfully integrated autoencoders with matrix factorization techniques.

Recent work has taken a further step by integrating heterogeneous network information. This allows models to move beyond simple feature learning and capture relationships more comprehensively. By extending nonlinear reasoning to graph structures and developing inductive deep learning frameworks, a unified architecture was converged that used graph representation learning and nonlinear matrix completion to process multi-source data and complex relationships. Liu et al. [118] used a multi-layered network method to integrate the similarity of multiple compounds and targets and proposed a new optimization framework called Multiple similarity Deep Walk-based Matrix Factorization (MDMF), which is used for CTI prediction. Mazzone et al. [119] proposed a data fusion method for CTI prediction, which is realized using the NXT fusion library. Their method extended traditional matrix factorization to realize the nonlinear inference of entity–relation graphs. In order to study how to extend the inductive learning framework to novel entities, Zabihian et al. [120] systematically compared two inductive depth network models. The IEDTI model adopted an interactive encoding strategy that learned joint representations directly from known CTIs. In contrast, the DEDTI model used a deep encoder–decoder architecture with symmetric feature extraction pathways to explicitly model bidirectional nonlinear relationships between drugs and targets. Experimental results on four benchmark datasets showed that DEDTI was always superior to IEDTI, and the AUCs on enzymes, ion channels, GPCRs and nuclear receptors reached 0.986, 0.986, 0.959 and 0.917, respectively. This performance advantage stemmed from DEDTI’s ability to more effectively separate entity features from interaction patterns, leading to stronger generalization in cold-start scenarios. This comparison showed that constructing symmetric and separate nonlinear mapping pathways within the neural matrix factorization framework was an effective way to improve model’s ability to generalize new compounds and new targets.

Matrix factorization methods excel at handling data sparsity by projecting compounds and targets into a shared latent space, a setting where classical supervised learning often struggles due to the lack of informative features for novel entities. This advantage is reflected in predictive performance. Models such as NRLMF and DEDTI have achieved AUC scores exceeding 0.96 on benchmark datasets, substantially outperforming classical methods like BLM under sparse interaction conditions [111,120]. Moreover, matrix factorization expands the prediction scope to cold-start scenarios. Interactions involving novel compounds or targets can be inferred through learned latent representations, whereas classical methods typically fail in such settings [112]. However, a key limitation of matrix factorization lies in their linear assumption. It captures interactions as linear combinations of latent factors, which may oversimplify complex biological relationships. Recent efforts to incorporate neural networks into matrix factorization frameworks have addressed this by introducing nonlinear mappings, but this comes at the cost of reduced interpretability. Another challenge is scalability. As the number of compounds and targets grows, factorization becomes computationally expensive, an issue less pronounced in classical methods that rely on fixed feature representations. Finding a balance between model complexity, interpretability, and computational efficiency remains an open challenge in this area.

### 4.3. Graph Topology-Based Inference Methods

Graph-based inference methods adopt a systems biology view for CTI prediction. They work by constructing a heterogeneous network that integrates multi-source data, including compound chemical similarities, target sequence homology, and known interactions. Specifically, compounds, targets, and diseases are represented as nodes in a network, with interactions as edges [121]. Techniques such as random walks [122], network propagation [123], or graph embedding [124] are then used to uncover complex relational patterns among entities. Compared to classical supervised learning methods and matrix factorization methods, graph topology-based inference methods take a holistic view of biological systems. They excel at uncovering indirect relationships hidden in multi-layered data, making them particularly useful for drug repositioning and novel target discovery [125]. Instead of relying on manual feature engineering, they let the network structure itself reveal the underlying biology through data-driven analysis, enabling more accurate and robust CTI predictions, particularly in sparse data scenarios.

The initial application of graph topology-based inference to CTI prediction can be traced back to Cheng et al. [126], who proposed a Network-Based Inference (NBI) framework. This method constructed a predictive model by utilizing compound and target similarity features within the topological space. Their method achieved impressive AUC scores of 0.975, 0.976, 0.946, and 0.838 for enzymes, ion channels, GPCRs, and nuclear receptors, respectively, across four benchmark databases. Later research subsequently introduced node-weighting and edge-weighting mechanisms, which improved the accuracy and stability of the predictions and elevated the AUC scores for GPCRs and kinase receptors to 0.981 and 0.976, respectively, in two databases [127]. The NBI algorithm was further extended by Alaimo et al. [128] with the introduction of the domain tuned-hybrid (DT-Hybrid) method. By integrating domain-specific knowledge, the method attained excellent AUC scores of 0.9995, 0.9973, 0.9995, and 1.000 for enzymes, ion channels, GPCRs, and nuclear receptors, respectively, across four benchmark databases.

With the increasing need to integrate different kinds of biological data, the research landscape has moved towards building complex networks that fuse information from multiple sources. The framework from Re et al. [129] provided a mathematical way to integrate chemical, biomolecular, and clinical information. It worked by taking networks that connect two different types of nodes, such as a compound–target network, and using the bipartite graph projection algorithms method to create a new network focused on just one type of node, like a compound–compound similarity network. These novel networks were then standardized or “normalized” using the normalized graph Laplacian to make them comparable and integrated into a final, comprehensive pharmacological network. This unified network then became the basis of more advanced algorithms. In the field of heterogeneous network analysis, Seal et al. [130] applied the Random Walk with Restart (RWR) algorithm to CTI prediction for the first time, which laid an important foundation for later diffusion-based methods. Yan et al. [123] developed the Label Propagation based on Multiple Information of Heterogeneous Networks (LPMIHN) algorithm. They first integrated the chemical structure of the compound, target protein sequences, and known interaction data to build a heterogeneous network. Then, using a label propagation mechanism, they allowed interaction information to flow back and forth between the compound subnetwork and the target subnetwork. This process helped to discover new potential compound–target relationships, achieving AUC scores of 0.9989, 0.9985, 0.9986, and 0.9960 for enzymes, ion channels, GPCRs, and nuclear receptors, respectively, across four benchmark databases. In another study, Wang et al. [131] used the Large-scale Information Network Embedding (LINE) method to extract topological features of compounds, targets, and disease from heterogeneous networks. The learned embeddings were then integrated to construct binary classification samples, which were then used to train a random forest (RF) classifier for CTI predictions. The LINE-RF model obtained showed strong predictive ability, with the AUC reaching 0.9349 and AUPR reaching 0.9016.

By operating directly on graphs, topology-based methods are good at capturing the overall structure of networks. However, they lack the ability to automatically learn complex, nonlinear features from raw data. This limitation has led to the emergence of GNNs, which will be discussed in the next section.

Graph-based methods offer the unique advantage of integrating heterogeneous data sources into a unified network, enabling the discovery of indirect relationships. They excel in scenarios requiring multi-source data integration and network-level insights, such as drug repurposing studies, where indirect relationships across heterogeneous networks are key. By propagating information through network structures, they can identify interactions involving low-degree nodes that have few known connections, a capability that classical and matrix factorization methods generally lack. Performance metrics support this strength. Methods like LPMIHN achieve near-perfect AUC scores on benchmark datasets, demonstrating their power in leveraging network topology [123]. However, their performance is often biased toward high-degree nodes, meaning well-studied compounds or targets, while nodes with few connections remain challenging to predict. Additionally, these methods typically rely on predefined network structures and lack the ability to learn complex nonlinear features directly from raw molecular data. This limitation may reduce predictive accuracy when subtle chemical features are critical. Consequently, graph-based methods are most effective when network connectivity provides a strong signal but may underperform when local chemical features dominate, a gap that GNNs later aimed to fill by learning both topological- and feature-based representations.

### 4.4. Deep Neural Network Frameworks

Deep neural networks represent a critical branch of machine learning. By applying multiple layers of nonlinear transformations, they automatically extract features and learn representations from raw data, eliminating the need for manual feature extraction [27]. These frameworks are built on artificial neural networks—computational models inspired by biological neural systems. These networks are made up of multiple layers of interconnected nodes, each layer building on the one before to gradually extract more meaningful features from the raw input. This allows the system to learn complex patterns autonomously, which has become a key advantage of deep learning. It enables these models to handle massive amounts of data effectively, achieving high performance and adapting well to novel situations.

Researchers have developed several specialized neural network architectures for different data structures. These includes CNNs [28], which use convolutional filters to efficiently deal with grid-like data by extracting local spatial features; RNNs [29] designed for sequential data by incorporating the memory of previous inputs; GNNs [132], which operate on graph-structured data to model relationships and interactions between entities; and the Transformer model [133], which uses a self-attention mechanism to process sequences in parallel, allowing it to capture long-range dependencies more effectively. These breakthroughs have also paved the way for deep learning in drug development, where it has proven particularly useful for predicting CTIs [29]. In this review, we group these methods into three broad architectural categories: sequence-based models (CNNs and RNNs), graph-based approaches (GNNs and network embedding), and Transformer-based architectures and discuss how each of these models has been applied to CTI prediction.

#### 4.4.1. Sequence-Based Representation Learning

In early approaches to CTI prediction with deep learning, compound molecules and target proteins were transformed into text-based representations. These methods, commonly known as sequence-based representation learning, used models like CNNs and RNNs to extract local patterns and capture sequential dependencies. For compound representation, CNNs have been proven to be effective at capturing local substructure patterns in SMILES strings, such as specific atom groupings or functional groups. For protein sequences, RNNs and their variants like BiLSTM are well suited to model sequential dependencies, capturing long-range residue interactions that are critical for folding and binding. These models are particularly effective when the predictive signal lies in local sequence patterns. For example, the study by Gupta et al. [134] demonstrated that recurrent neural networks containing LSTM units could accurately learn the grammatical structure of SMILES strings, enabling de novo molecular designs. Although this model was applied to molecule generation, it proved that deep learning models (such as RNNs and CNNs) can automatically extract effective features from one-dimensional sequence representations of compounds. This sequence-based representation learning approach was later widely adopted for drug–target interaction prediction: by employing convolutional or recurrent neural networks to mine the intrinsic patterns of molecular SMILES and protein sequences, it enables better prediction of the interaction probability or affinity between the two. Öztürk et al. [135] created a model with a two-branch CNN architecture that independently extracted features from SMILES strings and amino acid sequences. These feature representations were then fused and fed into a fully connected network to predict binding affinity. The model achieved strong AUPR scores of 0.714 on the Davis dataset and 0.788 on the KIBA dataset. To further refine the representation of protein sequences, Lee et al. [136] introduced a specialized DTI prediction model designed to capture local residue patterns characteristic of broad protein classes. By applying multi-scale convolutional operations to amino acid subsequences of varying lengths, their model could effectively identify functional motifs directly from raw sequences without relying on hand-crafted descriptors. This approach showed strong generalizability for DTI prediction, achieving an AUPR of 0.832 and AUC of 0.852 on an external validation dataset. Motivated by the success of such sequence-based modeling, subsequent research has developed hybrid architectures to further improve predictive performance. For instance, a novel prediction framework proposed by Zhang et al. [137] integrated knowledge graph embeddings with Bidirectional Long Short-Term Memory (BiLSTM) networks. This architecture leveraged the bidirectional contextual modeling of BiLSTM to effectively capture complex dependencies in biological sequences, leading to elevated prediction accuracy compared to conventional binary classifiers.

Sequence-based deep learning models, including CNNs and RNNs, have demonstrated strong performance by automatically extracting features from SMILES strings and protein sequences. Compared to classical and matrix factorization methods, sequence models offer superior performance on large-scale datasets where hand-crafted features are insufficient. However, they treat compounds and proteins as independent sequences, missing the topological relationships that are critical for understanding complex molecular interactions. This limitation motivated the development of GNNs, which explicitly model molecular graph structures.

#### 4.4.2. GNNs and Network Embedding

Basically, these sequence-based approaches work well for simpler prediction tasks where the patterns are obvious and local. However, compound–target interactions constitute complex heterogeneous networks, and GNNs excel at capturing the topological relationships between compounds and proteins and aggregating neighbor information through building specifically for graph-structured data, making them better suited for modeling the relational data that defines CTI prediction. Tsubaki et al. [138] proposed an end-to-end framework for CTI prediction that combined both GNNs and CNNs. The GNN branch learned graph-based representations of compounds, while the CNN branch extracted features from protein sequences. The integrated representations were then used for CPI prediction, and this method achieved impressive AUC scores of 0.971 for the *C. elegans* dataset. Shao et al. [139] proposed the DTIGCCN model, which employed spectral-based graph convolutional networks (GCNs) to extract features from compound and target expression profiles, coupled with a CNN to capture their latent relationships. The key advantage of this architecture lies in its ability to generate more refined and task-specific representations by fully exploiting the correlation structure between compounds and targets for prediction. Concurrently, graph neural network techniques have achieved significant breakthroughs. Meanwhile, graph neural network techniques have made rapid progress on both architectural depth and computational efficiency. Yang et al. [140] built a 27-layer deep GNN to capture multi-scale features of compounds and developed a novel high-performance visual interpretation method, Gradient-weighted Affinity Activation Mapping (Grad-AAM), to analyze deep learning models from the chemical perspective. This method achieved impressive AUC scores of 0.983 and 0.991 for the human dataset and *C. elegans* dataset, respectively. Zhang et al. [141] proposed a Simple-structured GNN (SS-GNN) model for accurate prediction of compound–target affinity. In this approach, protein–ligand interactions were represented as a single undirected graph based on distance thresholds, and protein covalent bonds were skipped to reduce computational complexity, leading to decreasing both the scale of the graph data and the associated computational cost.

Beyond molecular graphs, deep learning also pushed network-level inference forward. Shang et al. [142] proposed a multi-layer network representation learning method for CTI prediction. This approach integrated information across network layers to clean up noise and learn low-dimensional feature vectors for compounds and targets. They constructed multiple similarity networks based on different compound and target attributes, extracted features from each, and projected them into a shared interaction space for prediction. This design effectively enhanced the model’s robustness against noisy, heterogeneous data and improved its accuracy in predicting novel interactions. Chen et al. [143] introduced an interactive reasoning network based on a cascaded architecture. It built with embedding, encoding, interaction, and feature extraction layers to gradually abstract features and capture interactions at different levels. They successfully predicted and validated 22 Alzheimer’s disease-related targets with this model. In another study, Yu et al. [144] proposed the mtADENet framework that integrated multiple network inference techniques. The model significantly improved interpretability without sacrificing predictive stability. Further advancing the field, Zhang et al. [133] introduced DeepMAN, a model that was designed to enable dynamic interactions between compound and target representations through graph attention mechanisms and mutual attention networks. The DeepMAN model achieved excellent predictive performance, with an AUC of 0.956 and an AUPR of 0.959.

In summary, GNNs extend sequence-based models by explicitly modeling molecular graph structures, capturing topological relationships between atoms and bonds. This enables them to learn representations that reflect molecular connectivity, which is essential for understanding structure–activity relationships. Compared to sequence-based models, GNNs achieve superior performance on tasks where molecular topology is critical, such as predicting binding affinities for compounds with complex ring systems or multiple chiral centers [138,140]. Relative to graph topology-based inference methods, GNNs offer the advantage of learning nonlinear feature representations directly from raw molecular graphs rather than relying on predefined network structures. This allows GNNs to generalize better to novel compounds and to capture subtle chemical features that earlier graph-based methods missed. However, GNNs are computationally intensive and still face interpretability challenges. They are most suitable when the molecular structure provides a strong predictive signal and when large, annotated graph datasets are available.

#### 4.4.3. Transformer-Based Architectures

Originally designed for sequence modeling, the Transformer architecture is built around the self-attention mechanism. This design allows it to process data in parallel and capture long-range, global dependencies, making it highly suitable for diverse data types, including sequences and graphs. Unlike CNNs and GNNs, which rely on localized operations, the Transformer’s self-attention mechanism dynamically models global dependencies. This offers a distinct advantage in capturing the long-range interactions that are critical for complex biomolecular tasks and also drives the innovation in drug discovery modeling. Gao et al. [145] proposed GraphormerDTI, a graph Transformer-based model that represented molecular structures through a triple encoding of node centrality, spatial relationships, and edge attributes. The built-in structural inductive bias allowed it to be generalized for out-of-sample molecules and improved the performance on CTI prediction tasks. In another approach, Tang et al. [146] designed a model that used CNNs to extract features from compound SMILES strings and Transformers to handle protein sequences. These learned representations were then concatenated and fed into a multilayer perceptron to predict binding affinity, achieving impressive r2m index scores of 0.6703 on the Davis dataset and 0.7344 on the KIBA dataset.

Transformer-based architectures leverage self-attention mechanisms to capture long-range dependencies in both sequences and graphs. Unlike CNNs and GNNs, which rely on localized operations, Transformers dynamically model global dependencies, making them particularly effective for tasks requiring long-range context, such as predicting interactions involving large protein domains or complex molecular scaffolds. For compound SMILES strings, Transformers can capture global structural patterns beyond local neighborhoods. For protein sequences, they model interactions between distant residues without the sequential constraints of RNNs. Compared to sequence-based models and GNNs, Transformers offer improved performance on datasets with complex dependencies that span distant regions of sequences or graphs. They also benefit from parallel processing, enabling efficient training on large-scale datasets. However, Transformers are data hungry and computationally expensive, which limits their applicability when training data are scarce. In general, deep learning has pushed CTI prediction past the limits of traditional approaches, such as no more hand-crafted features, better cold-start performance, and the ability to handle high-dimensional sparse data.

As a result, prediction accuracy and model generalizability have both improved significantly. Among the various architectures, sequence-based models such as CNNs and RNNs are suited for tasks where local patterns in SMILES or protein sequences are predictive. They are computationally efficient but treat compounds and proteins independently, missing topological relationships. In contrast, GNNs are ideal when molecular topology is critical, such as for complex ring systems or scaffold hopping. They capture structural relationships explicitly but are computationally intensive and less interpretable. Transformers excel at capturing long-range dependencies, making them suitable for large protein domains or molecular scaffolds with distant interacting groups. Their self-attention enables global context capture but requires substantial data and computational resources. Despite these advancements, deep learning approaches for CTI prediction still face some challenges. First, they are highly data-intensive. Their performance relies heavily on large, high-quality datasets, which are often limited in many biological contexts. Second, the “black box” nature of deep learning models makes them difficult to interpret. This has been a real concern in drug discovery, where mechanistic understanding is critical. Techniques like attention mechanisms and saliency maps have made models more transparent, but interpretability remains an ongoing challenge. Third, deep neural networks are computationally expensive to train, which limits their accessibility. Hybrid approaches that integrate multiple modeling paradigms may provide a more balanced solution.

To provide a quantitative overview of the reported predictive performance across different methodological paradigms, we summarized the predictive performance of representative models as originally reported in the literature in Table 5 and Table 6. Table 5 focuses on models evaluated on the Yamanishi benchmark datasets, while Table 6 lists deep learning methods evaluated on independent benchmarks. It should be noted that direct numerical comparisons across different datasets, evaluation metrics, or data balance conditions are not strictly valid; the tables are intended to reflect the literature rather than to establish a leaderboard.

As shown in Table 5 and Table 6, the reported performance reveals a clear evolutionary trend across methodological paradigms. Early classical supervised learning methods, such as Yamanishi (2008) [6], established baseline AUCs of 0.84–0.90 on the imbalanced Yamanishi datasets. The subsequent introduction of bipartite local models by Bleakley (2009) [99] and, more importantly, the neighbor-based interaction profile inference by Mei (2013) [100] (BLM-NII) dramatically improved performance, achieving AUCs above 0.98 across all four target classes. This indicates that incorporating local network information and refining feature representation can push classical methods to near-optimal levels, at least for moderately sized datasets. Nevertheless, the dependence on hand-crafted features remains a limitation. Matrix factorization methods then introduced a different philosophy: handling data sparsity through latent factor models. Early KBMF achieved only moderate AUCs, but NRLMF and DEDTI variants raised the AUCs to 0.96–0.99, demonstrating that nonlinear mappings and regularization are critical for capturing complex interaction patterns. These methods excel in cold-start scenarios, yet their performance on the extremely sparse nuclear receptor dataset shows that pure factorization still struggles when positive examples are very few. Graph topology-based inference methods, represented by NBI, LPMIHN and DT-Hybrid, take a different route by leveraging pre-computed similarity networks. Their near-perfect AUC scores on the same imbalanced datasets highlight the extraordinary predictive power of network topology when the graph is sufficiently connected. However, this strength becomes a weakness for low-degree nodes, a trade-off not reflected in aggregate AUCs. Finally, deep learning methods, evaluated on larger human and *C. elegans* datasets rather than the Yamanishi benchmarks, achieve the highest AUCs (up to 0.995 by BridgeDPI), capitalizing on automatic feature extraction from raw sequences and graphs. Their data hungry nature, however, makes them ill-suited for small datasets like the nuclear receptor class, where even the best deep models are not directly comparable. Across all methods, the nuclear receptor dataset consistently poses the greatest challenge, reflecting the fundamental difficulty of prediction with extremely sparse interactions (only 90 known positive pairs). Overall, the progression from hand-crafted features to latent factors, network inference, and end-to-end learning illustrates a steady increase in predictive power but also reveals persistent trade-offs: interpretability vs. accuracy, sparsity handling vs. data hunger, and network richness vs. cold-start generalizability. No single method universally dominates; the choice should be guided by the specific task characteristics, data availability, and the relative importance of interpretability.

## 5. Applications of ML-Based CTI in Exploring Food Bioactive Compounds

Food bioactive compounds, such as phenolic acids, flavonoids, and alkaloids, are non-nutrient components that have exhibited biological activities beneficial for human health. However, for most of them, the targets and clear mechanisms remain unknown, which makes it hard to turn them into evidence-based functional products. To accelerate target discovery for food-derived compounds, researchers are extending ML-based CTI prediction methods from pharma to food science.

Food-derived compounds often occupy chemical spaces distinct from synthetic drugs, with complex scaffolds and multiple chiral centers. Traditional molecular fingerprints developed for drug-like molecules may not capture these features effectively. Several studies have addressed this by adopting fingerprint types or model architectures that are more sensitive to structural variations. In a study screening for xanthine oxidase inhibitors from a medicine-food homology library, researchers trained multiple fingerprint-algorithm classifiers and found that a topological–torsion random forest model achieved the best performance (AUC 0.992). This fingerprint type explicitly encodes bond torsions and topological features, which are particularly relevant for capturing the conformational flexibility of natural products [74]. Similarly, a study on Cyperus esculentus used a GCN-FG deep learning model to screen 152 active compounds against kinase targets. The graph convolutional network directly operated on molecular graphs, preserving topological relationships that substructure fingerprints might miss. The model achieved a mean squared error of 0.131 on the KIBA dataset and identified cyanidin chloride as a high-affinity MAP3K8 inhibitor, validated by molecular dynamics simulations [147].

Many food-derived compounds exert health benefits through synergistic effects on multiple targets rather than through a single high-affinity interaction. Conventional binary classification models are ill-suited for such scenarios. Hypergraph representation learning has emerged as a solution. By constructing herb–compound and disease–target hypergraphs, this approach models multi-layered connections that simpler methods miss. One study applied this strategy to successfully identify novel targets of natural compounds such as coumarin and progesterone for hormone-related disorders, inflammation, and cancer, with seven to eight out of the top ten predicted targets validated by the literature [148]. This method is particularly relevant for functional food research, where multi-target mechanisms are common.

Well-annotated interaction data for food-derived compounds are far less abundant than those for pharmaceutical drugs. Several studies have addressed this by leveraging transfer learning or integrating multiple data sources. The BioDeepNat model was developed to predict interactions between natural compounds and myocardial infarction-related targets. The model employed a deep neural network pre-trained on large-scale pharmaceutical datasets and fine-tuned on natural compound data, successfully identifying candidates such as E-resveratrol and kaempferol from common foods like grapes and tea [149]. Another study used a virtual screening pipeline integrating a fully connected neural network, ADMET characterization, and molecular dynamics simulations to identify food-derived dipeptidyl peptidase-4 inhibitors, yielding six candidates with significant therapeutic potential [150]. In addition, a study used random forest regression to predict the bioactivity of natural compounds against seven Alzheimer’s disease targets, identifying 166 candidates from food sources, including black walnut, ginger, and pepper, with subsequent in vitro validation [151]. These examples illustrate that, even when experimental data are limited, computational strategies such as transfer learning, high-throughput virtual screening, and careful feature selection can enable effective CTI prediction for food compounds.

Beyond direct CTI prediction, recent studies have begun incorporating multi-omics data to improve the functional relevance of predictions. Food-derived compounds undergo extensive metabolism, and their bioactive metabolites may differ from the parent compounds. Metabolomics can profile the compounds that actually reach systemic circulation. For example, a study on dietary polyphenols integrated metabolomics data to identify circulating metabolites and then used ML-based target prediction to map these metabolites to vascular disease-related pathways [152]. Similarly, transcriptomics data from food intervention studies can inform target prioritization. One study combined CTI prediction with gene expression profiles from patients with myocardial infarction, using the BioDeepNat model to prioritize targets that were both computationally predicted and differentially expressed in the disease [149]. Moreover, the field of foodomics has established frameworks for integrating metabolomics, transcriptomics, and proteomics data with machine learning to uncover bioactive compounds and their molecular targets [153]. Knowledge graphs that link food composition, chemical bioactivity, and disease associations have also emerged as powerful tools for hypothesis generation [154]. For example, a quantitative metabolomics-driven workflow integrated untargeted metabolomics data with molecular docking to predict protein targets of dietary metabolites, revealing that gut metabolites derived from blueberry consumption predominantly interact with carbonic anhydrase family proteins involved in acid–base regulation and bone metabolism [155]. Another study developed a multi-target screening pipeline that combined ML-based bioactivity prediction with physiologically based pharmacokinetic modeling to design flavonoid-based functional products for metabolic dysfunction-associated steatotic liver disease [156]. Such integrative approaches are still in their infancy but hold great potential for evidence-based functional food development by bridging the gap between computational predictions and biological activity.

Taken together, these studies illustrate that the choice of ML method and feature representation should align with the specific characteristics of food-derived compounds. Structural diversity calls for topology-sensitive fingerprints or graph-based models. Multi-target effects benefit from hypergraph- or network-based approaches. Data scarcity can be mitigated by transfer learning or the integration of multiple data sources. Food-omics integration, while still in its early stages, offers a path toward more physiologically relevant predictions. By screening food compounds against disease-related targets, these methods not only accelerate discovery but also lay the mechanistic groundwork for evidence-based functional foods.

## 6. Discussion

The identification of CTIs is a fundamental task in drug discovery and is also of increasing importance for understanding the mechanisms of food-derived bioactive compounds. In this review, we have systematically summarized recent advances in ML-based CTI prediction, including commonly used databases, feature extraction methods, and typical modeling strategies, with a particular focus on the methodological evolution from classical supervised learning to deep neural networks.

Classical supervised learning methods, while highly interpretable and effective on small datasets, rely heavily on predefined molecular fingerprints and protein descriptors and often struggle in cold-start scenarios where novel compounds or targets lack existing interaction records. Matrix factorization methods introduced a paradigm shift by learning latent representations directly from interaction data, handling data sparsity effectively, though their linear assumptions may oversimplify complex biological relationships. Graph topology-based inference methods take a systems biology perspective, integrating heterogeneous data sources into unified networks to uncover indirect relationships, yet they can suffer from bias toward high-degree nodes. Deep neural network frameworks have set new state-of-the-art benchmarks by automatically learning hierarchical representations from raw sequences and molecular graphs, eliminating the need for manual feature engineering, but they remain data hungry, computationally expensive, and often function as “black boxes” with limited interpretability.

A central challenge in reviewing this field is the fair comparison of model performances across studies. While numerous models have reported high accuracy, AUC, or AUPR scores on benchmark datasets, direct numerical comparisons must be interpreted with considerable caution. Evaluation metrics vary across studies, with AUC and AUPR responding differently to class imbalances. AUC measures the model’s ability to discriminate between positive and negative classes across all classification thresholds, providing an overall assessment of ranking performance. AUPR, on the other hand, focuses on the performance on the positive class by evaluating the trade-off between precision and recall, making it particularly informative when positive instances are rare [157,158]. Even when the same metric is used, variations in data preprocessing, train–test splitting strategies, and negative sample construction can substantially influence reported performance. The choice of validation protocol, random splitting versus cold-start validation, also dramatically affects results. These methodological heterogeneities mean that a model reporting a higher score is not necessarily superior to another with a slightly lower score; differences may reflect experimental choices rather than true algorithmic advances. The field would benefit from standardized evaluation protocols and the adoption of cold-start validation as a complementary benchmark.

Beyond these evaluation-specific issues, several broader methodological challenges warrant attention. Dataset bias remains a concern, as most public databases are skewed toward well-studied targets and drug-like compounds, limiting model generalizability to understudied areas such as food-derived compounds. Negative sample construction lacks standardization, with different studies employing varied heuristic strategies that can substantially influence performance and hinder cross-study comparability. Reproducibility is also a persistent challenge, as many studies do not share code, data splits, or detailed experimental settings, making independent validation difficult. Addressing these challenges will require concerted efforts toward standardized protocols and transparent reporting.

Food-derived compounds exhibit distinct chemical characteristics compared to synthetic drugs, including high structural diversity, complex natural product scaffolds, and frequent chiral centers. These features may not be adequately captured by models trained predominantly on drug-like molecules. Moreover, the mechanisms of action for food-derived compounds often involve multi-target synergistic effects rather than single-target interactions, yet most current models are designed for binary interaction prediction. The scarcity of well-annotated interaction data for food-derived compounds further complicates model training and validation, as databases like FooDB and TCMSP, while valuable, are less comprehensive than pharmaceutical databases. Bioavailability and metabolic transformation add another layer of complexity, as a compound showing promising in silico interactions may have low oral bioavailability or be extensively metabolized.

Future efforts should prioritize the development of food-specific benchmark datasets, the integration of structural biology tools, and the design of interpretable models that can bridge computational predictions with experimental validation. By providing a structured synthesis of current methodologies and a critical analysis of their application to food science, this review aims to serve as a resource for researchers in both drug discovery and functional food research.

## 7. Conclusions

This review systematically summarized recent advances in ML-based CTI prediction, with a focus on the methodological evolution from classical supervised learning to deep neural networks. The four-paradigm classification framework proposed here captures the progression of the field and highlights the complementary strengths and limitations of each approach. By integrating this framework with an analysis of feature extraction strategies and a focused examination of applications to food-derived bioactive compounds, this review offers a structured resource for researchers in both drug discovery and functional food science. Looking forward, addressing the challenges of data scarcity, model interpretability, and the unique characteristics of food-derived compounds will be essential to advance the field.

## Figures and Tables

**Table 1:** Summary of public databases for CTI prediction.

Category	Database	Key Content	Key Features
Comprehensive Pharmaceutical Databases	DrugBank	FDA-approved drugs, investigational compounds, drug–drug and drug–food interactions	Over 200 standardized data fields; detailed interaction mechanisms
	KEGG DRUG	Drug structures, target networks, metabolic enzyme information	Integrated with KEGG pathways; organized by ATC codes
	ChEMBL	Bioactivity data (IC50, Ki), ADMET properties	5.4 million bioactivity data points; bridges chemical and biological information
Drug–Target Interaction Databases	STITCH	CTIs	Confidence scores; integrates experimental and text-mined data
	BindingDB	Binding affinities (Kd, Ki)	1.13 million binding constants; 7020 target proteins
	SuperTarget	Drug–target interactions, PPIs, adverse effects	Multi-dimensional network analysis; supports drug repurposing
	MATADOR	Direct binding and indirect regulatory relationships	Manually curated with text mining
Protein and Biomolecular Databases	UniProt	Protein sequences, functional annotation, subcellular localization	Swiss-Prot (manual) and TrEMBL (automatic)
	BRENDA	Enzyme kinetic parameters (Km, kcat)	Data from 140,000 papers; comprehensive enzyme information
	Pfam	Protein families and domains	Hidden Markov model-based classification; 13,000 families
	BioGRID	PPIs, genetic interactions, PTMs	Curated interaction data for network analysis
Chemical Compound Databases	PubChem	Chemical structures, physicochemical properties, bioactivity	119 million compounds; standardized screening data
	ZINC-22	3D structures of commercially available compounds	200 million molecules; substructure search
Food Ingredients Related Databases	FooDB	Food ingredient composition and bioactivity	70,000 food ingredients; constantly updated
	TCMSP	Herbal ingredients, targets, pharmacokinetics	Traditional Chinese medicine systems pharmacology
Other Databases	SIDER	Adverse drug reactions	6000 drugs; 140,000 side effect records
	FAERS	Real-world adverse event reports	FDA adverse event data
	DCDB	Drug combinations and synergies	1300 combinations; mechanism information

**Table 2:** Software/server and its calculated fingerprints.

Software/Server	Type	Main Supported Fingerprint/Descriptor Types
CDK 2.12 [53]	Open-source Java Library	Dictionary-based fingerprints, Circular fingerprints (ECFP, FCFP), Topological fingerprints, Pharmacophore fingerprints, Heteroatom skeletons, numerous Physicochemical descriptors.
PaDEL-Descriptor 2.21 [54]	Desktop/Command-line Software	Dictionary-based fingerprints (PubChem, MACCS, Klekota-Roth), Circular fingerprints, Topological fingerprints (Atom Pairs, Torsions), Substructure-based fingerprints, Autocorrelation descriptors, etc., totaling over 1000 types.
JCompoundMapper [55]	Java Tool Library	Topological fingerprints (2D Atom Pairs, Shortest Path, CATS), Edge-based fingerprints, Heterogeneous environment fingerprints.
ChemoPy [56]	Python Package	Dictionary-based fingerprints (PubChem, MACCS), Physicochemical descriptors, Topological descriptors, Geometrical descriptors, MOE-type descriptors.
RDKit v2026.03.1	Open-source Toolkit	Dictionary-based fingerprints (MACCS), Circular fingerprints (Morgan fingerprints, equivalent to ECFP), Topological fingerprints (Atom Pairs, Torsions), Hash-based fingerprints.
Scikit-fingerprints v1.12.0 [57]	Python Package	Built on RDKit, provides a unified interface for over 30 molecular fingerprints.
ChemDes [58]	Online Server	Integrates multiple open-source tools (RDKit, CDK, Pybel), can calculate 59 types of molecular fingerprints and 3679 types of molecular descriptors.
OpenBabel 3.1.1 [59]	Open-source Software	Several classic fingerprints like FP2, FP3, FP4, MACCS.
MolAICal 2.0a [60]	Software Tool	Supports fingerprint-based and 3D structure-based molecular similarity comparison and virtual screening.
ProLIF [61]	Python Package	Used to generate IFPs, not structural fingerprints.

**Table 3:** Servers and calculated sequence-derived features.

Software/Server	Main Supported Fingerprint/Descriptor Types
PROFEAT [75]	Amino acid composition; Dipeptide composition; Normalized Moreau–Broto autocorrelation; Moran autocorrelation; Geary autocorrelation; Composition; Transition; Distribution; Sequence-order-coupling number; Quasi-sequence-order descriptors
Pse-in-One [76]	Basic k-mer; Auto covariance; Cross covariance; Auto-cross covariance; Parallel correlation PseAAC; Series correlation PseAAC; General parallel correlation PseAAC; General series correlation PseAAC
IFeature 1.0.0 [77]/iFeatureOmega [78]	Amino acid composition; Dipeptide composition; Tripeptide composition; AAIndex-based descriptors; BLOSUM/PAM matrix encoding; PSSM-composition; Pseudo-position-specific scoring matrix; Type I/II PseAAC; Amphiphilic PseAAC; Quasi-sequence-order descriptors; Sequence-order-coupling number; Shannon entropy; Mutual information; De novo deep learning-based features; Feature fusion and analysis capabilities
Pfeature 1.4 [79]	Composition-based; Binary profile patterns; Autocorrelation descriptors; Evolutionary-based (PSSM composition); Structural propensity-based; Customizable hybrid feature set
RepDNAv1.1.4 [80]	Nucleic acid composition; Dinucleotide composition; Trinucleotide composition; Autocorrelation; PseKNC; Chemical property-based features; Electron-ion interaction pseudopotentials

**Table 4:** Comparison of computational methodologies.

Category	Representative Algorithms	Input Data Type	Advantages	Limitations
Classical Supervised Learning	SVM, RF, BLM	Fingerprints, Descriptors	High interpretability; Easy to implement; Good for small datasets.	Relies on hand-crafted features; Cannot handle cold-start problem well.
Matrix Factorization	NRLMF, KBMF, MSCMF	Interaction Matrix	Handles data sparsity effectively; Good for large-scale screening.	Limited nonlinear capability (unless neural); Linear assumptions.
Graph Topology-based Inference	RWR, Label Propagation	Heterogeneous Networks	Captures global network structure; No need for negative samples.	Ignores local chemical features; Biased towards high-degree nodes.
Deep Neural Frameworks	CNN, GNN, Transformer	Raw Sequences, Molecular Graphs	End-to-end learning; SOTA performance; Handles complex nonlinear patterns.	“Black box” (low interpretability); Data hungry; High computational cost.

**Table 5:** Performance of representative models on the Yamanishi benchmark datasets.

Category	Model	Metric	Enzyme	Ion Channel	GPCR	Nuclear	Refs.
Classical Supervised	Yamanishi	AUC	0.904	0.851	0.899	0.843	[6]
	BLM	AUPR	0.841	0.813	0.667	0.612	[99]
	BLM-NII	AUC	0.988	0.990	0.984	0.981	[100]
	Bigram-PSSM	AUC	0.948	0.889	0.872	0.869	[101]
	Ding	AUPR	0.899	0.929	0.821	0.655	[96]
	PDTPS	Accuracy	0.977	0.931	0.868	0.878	[104]
	SELF-BLM	AUC	0.859	0.941	0.914	0.799	[97]
Matrix Factorization	KBMF	AUC	0.832	0.799	0.857	0.824	[110]
	MSCMF	AUPR	0.894	0.937	0.773	0.673	[109]
	NRLMF	AUC	0.966	0.964	0.930	0.851	[111]
	DEDTI	AUC	0.986	0.986	0.959	0.917	[120]
Graph Topology	NBI	AUC	0.975	0.976	0.946	0.838	[126]
	DT-Hybrid	AUC	0.9995	0.9973	0.9995	1.000	[128]
	LPMIHN	AUC	0.9989	0.9985	0.9986	0.9960	[123]
	LINE-RF	AUC	0.9349 (LINE-RF reported an overall AUC)				[131]

**Table 6:** Performance of deep learning models on independent benchmarks.

Model	Dataset	Metric	Performance	Refs.
DeepDTA	Davis	AUPR	0.714	[135]
	KIBA	AUPR	0.788	
DeepConv-DTI	external val.	AUPR/AUC	0.832/0.852	[136]
GNN-CNN	C. elegans	AUC	0.971	[138]
MGraphDTA	Human	AUC	0.983	[140]
	C. elegans	AUC	0.991	
DeepMAN	benchmark	AUC/AUPR	0.956/0.959	[133]
GraphormerDTI	multi	AUC	0.802–0.926	[145]
TC-DTA	Davis	r^2^ (m)	0.6703	[146]
	KIBA	r^2^ (m)	0.7344	

## Data Availability

No new data were created or analyzed in this study. Data sharing is not applicable to this article.

## Abbreviations

The following abbreviations are used in this manuscript:

CTIs	Compound–target interactions
ML	Machine learning
HTS	High-throughput screening
CNNs	Convolutional Neural Networks
LSTM	Long Short-Term Memory
ML-CNN	ML algorithm-based Convolution Neural Network
GNNs	Graph Neural Networks
KEGG	Kyoto Encyclopedia of Genes and Genomes
ATC	Anatomical Therapeutic Chemical
STITCH	Search Tool for Interacting Chemicals
UniProt	Universal Protein Resource
BRENDA	Braunschweig Enzyme Database
AD	Alzheimer’s disease
BioGRID	Biological General Repository for Interaction Datasets
SIDER	Side Effect Resource
FAERS	FDA Adverse Event Reporting System
DCDB	Drug Combination Database
FooDB	Food Database
TCMSP	Traditional Chinese Medicine Systems Pharmacology
MACCS	Molecular ACCess System
ECFP	Extended Connectivity Fingerprint
QSAR	Quantitative Structure–Activity Relationship
PseAAC	Pseudo amino acid composition
PSSM	Position-Specific Scoring Matrices
HMM	Hidden Markov Model
PPIs	Protein–protein interactions
RNNs	Recurrent Neural Networks
SVM	Support Vector Machines
AUPR	Area Under the Precision-Recall Curve
BLM	Bipartite Local Model
AUC	Area Under the Curve
PDTPS	Predicting Drug Targets with Protein Sequence
KBMF	Kernelized Bayesian Matrix Factorization
MSCMF	Multiple Similarities Collaborative Matrix Factorization
NRLMF	Neighborhood Regularized Logistic Matrix Factorization
SPLCMF	Self-paced learning with collaborative matrix factorization
MDMF	Multiple similarity Deep Walk-based Matrix Factorization
NBI	Network-Based Inference
DT-Hybrid	Domain tuned-hybrid
RWR	Random Walk with Restart
LPMIHN	Label Propagation based on Multiple Information of Heterogeneous Networks
LINE	Large-scale Information Network Embedding
RF	Random forest
BiLSTM	Bidirectional Long Short-Term Memory
CPIs	Compound-protein interactions
GCN	Graph convolutional networks
Grad-AAM	Gradient-weighted Affinity Activation Mapping
SS-GNN	Simple-structured GNN
BioDeepNat	Bioinformatics-integrated deep neural analysis of NCs
FCNN	Fully connected neural network
XO	Xanthine oxidase
